# A Large-Scale Method to Measure the Stoichiometries of Protein Poly-ADP-Ribosylation

**DOI:** 10.1021/acschembio.5c00817

**Published:** 2026-02-26

**Authors:** Peng Li, Yajie Zhang, Chiho Kim, Yonghao Yu

**Affiliations:** † Department of Biochemistry, University of Texas Southwestern Medical Center, Dallas, Texas 75390, United States; ‡ Department of Molecular Pharmacology and Therapeutics, Columbia University Vagelos College of Physicians and Surgeons, New York, New York 10032, United States

## Abstract

Poly-ADP-ribosylation (PARylation) is a reversible post-translational modification that occurs in higher eukaryotes. While thousands of PARylated substrates have been identified, the specific biological functions of most PARylated proteins remain elusive. PARylation stoichiometry is a critical parameter to assess the potential functions of a PARylated protein. Here, we developed a large-scale strategy to measure the stoichiometries of protein PARylation. By integrating chemically mild cell lysis conditions, boronate enrichment, and carefully designed titration experiments, we were able to determine the PARylation stoichiometries for a total of 235 proteins. Importantly, this approach enables the capture of all PARylation events, regardless of their amino acid acceptor linkages. We revealed that PARylation occupancy spans over 3 orders of magnitude. However, most PARylation events occur at low stoichiometric values (median 0.58%). Notably, we observed that high-stoichiometry PARylation (>1%) predominantly targets proteins involved in transcription regulation and chromatin remodeling. Thus, our study provides a system-scale, quantitative view of PARylation stoichiometries under genotoxic conditions, which serves as an invaluable resource for future functional studies of this important protein post-translational modification.

## Graphical abstract

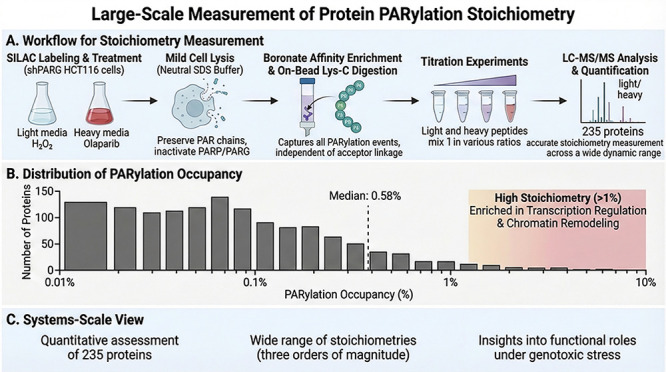

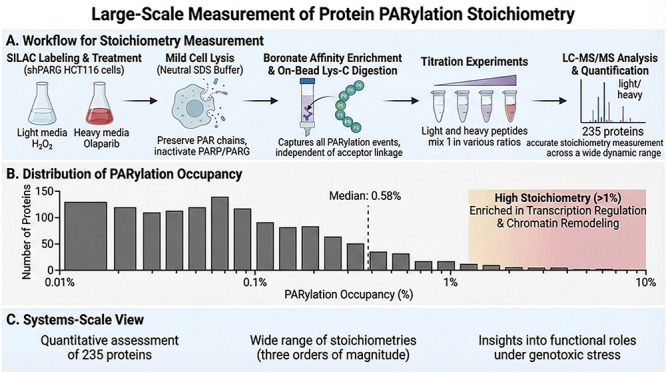

## Significance

Poly-ADP-ribosylation (PARylation) is a dynamic, heterogeneous protein modification that is critically involved in the DNA damage response. Although the PARylation stoichiometry is a key functional determinant for PARylated proteins, the PARylation occupancy of these modified proteins is largely unknown. In this study, we developed a large-scale proteomic approach for quantifying the stoichiometry of protein PARylation. By integrating optimized boronate affinity enrichment with SILAC-based titration, we systematically determined the PARylation occupancies for 235 proteins. Notably, PARylation targets with high stoichiometric levels are enriched for transcription factors and chromatin remodelers, pointing to the role of PARylation in regulating nuclear processes beyond DNA repair. This PARylation stoichiometry database provides a foundational resource for elucidating the functional impact of PARylation on a large variety of pathophysiological mechanisms.

## Introduction

Mammalian cells are continuously exposed to genotoxic stress and, as a result, have evolved sophisticated mechanisms to detect and signal the presence of damaged DNA, thereby facilitating efficient DNA repair processes. One of the earliest cellular responses following exposure to genotoxic stress occurs through the reversible PTM poly-ADP-ribosylation (PARylation), which is catalyzed by a family of enzymes known as poly­(ADP-ribose) (PAR) polymerases (PARPs).^1^ Of all of the PARP family members, PARP1 exhibits the highest expression level and has robust enzymatic activity. PARP1 accounts for approximately 90% of the PAR polymer formed under genotoxic conditions.^2^ PARP1 utilizes intracellular NAD^+^ to synthesize PAR polymers, which are covalently linked to proteins via the attachment to a variety of amino acid acceptors, including Glu, Asp, Ser, Tyr, Cys, and Lys.^3–5^

The synthetic lethality between PARP1 inhibition and *BRCA1/2* deficiency provides the mechanistic foundation for the development of PARP1 inhibitors for the treatment of human malignancies.^6^ Indeed, PARP1 inhibitors are successful in the clinic, with four PARP1 inhibitors (i.e., Olaparib, Niraparib, Rucaparib, and Talazoparib) approved by the FDA to treat *BRCA* ^mut^ breast, ovarian, prostate, and/or pancreatic cancers.^7–10^ Despite the tremendous progress in these PARP1 inhibitors, their fundamental mechanisms of action remain incompletely understood.

Auto-PARylated PARP1 functions as a scaffold that facilitates the recruitment of various DNA repair machineries, such as base excision repair, nucleotide excision repair, and double-strand break repair, often through their PAR-binding domains.^11–13^ While the initial focus of this field centered on the role of PARP1 in DNA damage repair, our understanding of the functions of PARylation signaling has significantly expanded in recent years. Notably, new findings point to the diverse functions of PARylation signaling in chromatin regulation, transcription, RNA biology, metabolism, and viral infections.^14,15^ Furthermore, the recent development of PARylation proteomic technologies has led to the identification of many PARylated proteins, which, in turn, greatly facilitates the functional studies of this important protein modification.^16–26^

Stoichiometry (the fraction of a given protein that is modified at a given time) is a critically important parameter when assessing the potential function of a post-translationally modified protein.^27^ For example, the transcription factor FOXO3a is known to be phosphorylated by a Ser/Thr kinase Akt, at three critical residues (i.e., T32, S253, and S315).^28^ When Akt is inhibited, FOXO3a is dephosphorylated and is subsequently translocated into the nuclei to mediate the transcription of a variety of pro-apoptotic genes. It has been shown that the stoichiometry of the phosphorylation at the three aforementioned residues governs the relative distribution of FOXO3a in the cytoplasm vs nuclei, and in so doing, provides a mechanism for fine-tuning its transcriptional output.^28^ Similarly, in the context of PARylation, it has been shown that PARP1 covalently modifies p53 on three evolutionarily conserved residues (i.e., E255, D256, and E268). PARylation of p53 blocks its interaction with the nuclear export receptor, CRM1, resulting in the nuclear accumulation of p53 and initiation of the p53-dependent apoptosis program.^29^ It is conceivable that PARylation stoichiometry could be a key factor that regulates the nuclear-cytoplasmic distribution of p53. PARylation stoichiometry, therefore, is a key parameter when assessing its functional role in regulating protein–protein interactions, subcellular distribution, and protein activity.

While several biochemical methods have been employed to measure stoichiometries of other PTMs (e.g., phosphorylation), these methods are not applicable to PARylation stoichiometry measurement. This is primarily due to the labile, heterogeneous, and low-abundance nature of PARylation.^30,31^ In particular, PARylation is highly dynamic and is known to be under precise spatiotemporal control.^32^ Previously, we developed a large-scale mass spectrometric approach for the site-specific characterization of the Asp-/Glu-PARylated proteome. Coupled with SILAC (Stable isotope labeling by amino acids in cell culture) labeling, this approach allowed the quantification of Asp-/Glu-PARylation events under different cellular states (i.e., control vs PARP1-inhibited).^16^ However, these relative changes alone cannot be used to determine PARylation stoichiometries. For instance, a 2-fold downregulation of a PARylation site, as measured in a SILAC experiment, could result from a change in stoichiometry, e.g., from 0.2 to 0.1% or from 100 to 50%. These two scenarios likely have very different biological implications.

In this study, we developed a large-scale approach for the measurement of PARylation stoichiometries. By combining chemically mild cell lysis conditions, high efficiency boronate enrichment of the PARylated proteins, and carefully designed titration experiments, our approach allowed the determination of PARylated stoichiometries of 235 proteins under oxidative DNA damage conditions. Importantly, this approach encompasses all PARylation events occurring on different amino acid side chains. Our findings reveal a wide range of PARylation occupancy spanning 3 orders of magnitude (ranging from 35.48 to 0.01%). However, it is noteworthy that the majority of PARylation events occur at low stoichiometries, with a median value of 0.58%. Importantly, our bioinformatic analysis indicates that proteins with high PARylation occupancy (stoichiometry >1%) primarily comprise transcription regulators and chromatin remodelers. This work not only provides valuable insights into the principles of PARylation-dependent signaling but also seeds the future functional studies of this important protein modification.

## Results

### Overall Strategy for Determining Global PARylation Stoichiometries

PARylation is a labile, heterogeneous, and low-abundance PTM. Recent studies have shown that PARylation can occur on multiple amino acid acceptors, including Glu, Asp, Ser, Tyr, Cys, and Lys.^4,16–20,22,23,33^ We previously developed an integrated strategy toward the site-specific analysis of the cellular Asp-/Glu-PARylated proteome. The bond between ADP-ribose and an Asp/Glu residue constitutes a high-energy “hemiacetal ester”, and is chemically unstable.^16,34,35^ Furthermore, PARylation could also be aberrantly added and removed by PARP1/PARG during the cell lysis step. In our approach, we used a chemically mild lysis buffer (e.g., neutral SDS buffer) to deactivate the PARP/PARG enzymes and to universally preserve the PAR chains. Importantly, the SDS buffer system is fully compatible with the subsequent boronate enrichment procedures.^16^ This approach was originally developed for the analysis of the cellular Asp-/Glu-PARylated proteome. However, it is important to note that other PARylation linkages are much more stable (e.g., Ser-PARylation),^19^ and are also preserved under these lysis conditions.^36^ The cell lysis conditions, therefore, represent a lysis buffer that is broadly applicable for proteomic analyses of protein PARylation.

The boronate enrichment strategy leverages the formation of ester bonds between boron and a 1,2-*cis*-diol moiety within ADP-ribose. Here, we further evaluated the enrichment efficiency of the boronate system. We found that a single round of boronate enrichment largely abolished the PAR signal in whole-cell lysates. Following the second round of boronate pull-down, no detectable PARylation was observed in the supernatant (Figure 1A), indicating complete recovery of the PARylated proteins from crude cell lysates by the boronate beads. Other methods (e.g., macrodomain Af1521) have been developed for the enrichment of PARylated proteins.^37,38^ However, the recovery efficiencies of these methods have not been systematically evaluated. Furthermore, macrodomain Af1521 has intrinsic hydrolase activity against Asp-/Glu-PARylation, and it also displays unequal affinity to PARylation with different amino acid linkages.^35,39^ It is important to note that enrichment of PARylated proteins by boronate beads is based on chemical bond formation (between the cis-diol and boronate) rather than protein-ADP-ribose binding (e.g., Af1521). The boronate beads, therefore, are uniquely characterized by their high enrichment capacity, their tolerance for the SDS washing buffer, their broad compatibility with different PARylation-amino acid linkages, and, finally, on-bead enzymatic digestion.

**Figure 1:**
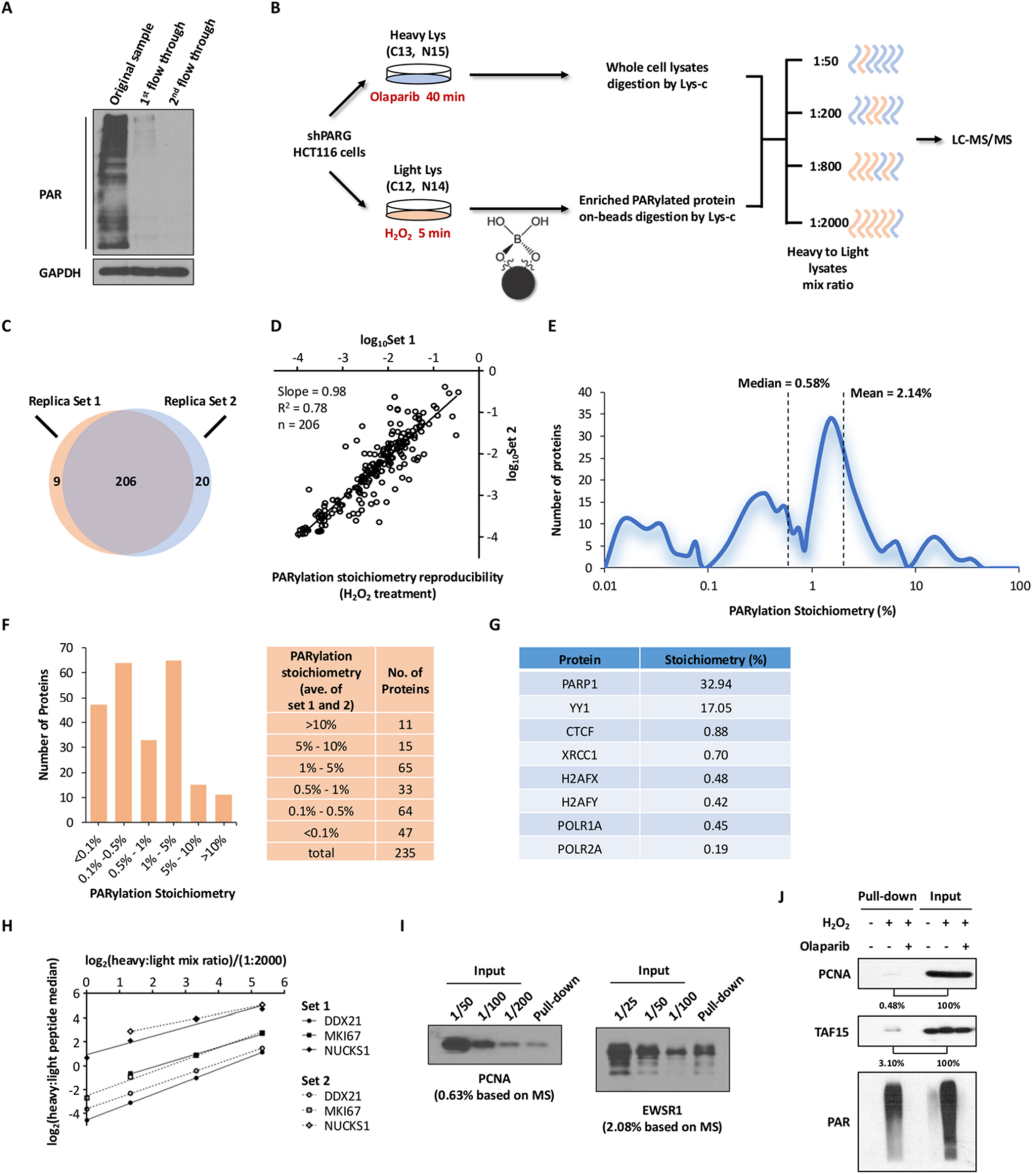
Global PARylation stoichiometry is low but spans a large dynamic range. (A) PARylated proteins were quantitatively adsorbed from crude cell lysates using BAB. Cell lysates (shPARG HCT116 cells treated with 2 mM H_2_O_2_) were incubated with BAB twice. The extent of PARylation in the original sample and in two flow-through fractions was assessed using immunoblotting assays with an anti-PAR antibody. (B) Flowchart illustrating the overall strategy for determining global PARylation stoichiometries. Light shPARG HCT116 cells were treated with H_2_O_2_ (2 mM, 5 min) to induce protein PARylation, while heavy cells were treated with Olaparib (1 μM, 40 min) to suppress basal PARylation levels. Enriched PARylated proteins from light cells were subject to on-beads digestion. Light and heavy peptides were then mixed at different protein ratios in the whole-cell lysates. (C) Biological replicate experiments were conducted using SILAC HCT116 shPARG cells to determine PARylation stoichiometry. Set 1 identified 215 PARylated proteins, while Set 2 identified 226 PARylated proteins. (D) Reproducibility of PARylation stoichiometry between biological replicates. (E) Overall distribution of protein PARylation stoichiometries (*n* = 235). (F) Distribution of PARylation stoichiometry for grouped PARylated proteins (*n* = 235). (G) PARylation stoichiometry of previously well-studied PARP1 substrates. (H) Dynamic profiles of the median ratio between heavy and light at various SILAC cell lysate mix ratios for individual proteins were compared between biological replicates. (I) Validation of PARylation stoichiometry for PCNA and EWSR1. HCT116 shPARG cells treated with H_2_O_2_ (2 mM, 5 min) were lysed. Lysates were subject to boronate beads pull-down, and the PARylated proteins were eluted as described before. The eluted eluates and the serially diluted cell lysate input were analyzed by immunoblotting to validate PARylation stoichiometry. (J) Validation of PARylation stoichiometry for PCNA and TAF15 in wild-type cells. Wild-type HCT116 cells treated with H_2_O_2_ (2 mM, 5 min) and/or Olaparib (1 μM, 40 min) were lysed. Lysates were subject to boronate beads pull-down, and the PARylated proteins were eluted as described before. Input lysates (Total) and boronate-enriched pull-down fractions (PARylated proteins) were analyzed by immunoblotting with antibodies against PCNA, TAF15, and PAR to validate PARylation stoichiometry.

We leveraged the excellent enrichment efficiency of the boronate system to develop a strategy toward measuring global PARylation stoichiometries (Figure 1B). We first generated shPARG HCT116 cells and labeled them with stable isotopes (light Lys and heavy Lys, respectively). The light cells were treated with H_2_O_2_, which induced oxidative DNA damage and PARP1 activation.^16^ The heavy cells were treated with Olaparib to completely abolish any basal PARylation. We isolated the PARylated proteins from the light cell lysates using the boronate affinity beads.^16^ The beads were extensively washed using the SDS buffer, and the PARylated proteins were digested on-beads using Lys-C. On the other hand, the lysates from heavy-labeled cells were directly digested using Lys-C without boronate enrichment. After digestion, aliquots containing light or heavy peptides were combined and analyzed by LC-MS/MS experiments. Because the boronate beads completely recover the PARylated proteins from the lysates, we reasoned that the ratio of the light and heavy versions of a PARylated protein determined from these SILAC experiments represents its PARylation stoichiometry.

Because PARylation is of very low abundance, it is likely that the PARylated version of a protein presumably represents a very small fraction of the total pool of that protein. The Orbitrap mass detector offers a dynamic range of approximately 5000.^40^ It is important to note that the light-heavy peptide pairs need to have a suitable ratio that is amenable to the dynamic range of the subsequent mass spectrometric detection. Toward this, we performed a series of titration experiments where we used different mixing ratios for the light/heavy samples. For example, we performed on-bead Lys-C digestion of the PARylated proteins isolated from 10 mg of light cell lysates. Separately, we performed Lys-C digestion of 5 μg of heavy cell lysates. The mixing of the peptides resulted in a 1:2000 heavy-to-light mix ratio. Similarly, a 1:50 heavy-to-light mix ratio was obtained when the same quantity (10 mg) of light peptides was mixed with a digestion solution of 200 μg of heavy lysates. We performed a series of experiments, employing the different heavy/light mixing ratios ranging from 1:2000 to 1:50 (Figure 1B). The resulting ratios of the heavy/light peptides were then converted back by using the original mixing ratios to determine the PARylation stoichiometry of a PARylated protein.

### Global PARylation Stoichiometries Span a Wide Dynamic Range

To precisely quantify the stoichiometries of PARylation induced by genotoxic stress, we conducted a large-scale, quantitative MS analysis as described in Figure 1B. To implement our strategy, we utilized a curated list of PARylated proteins with known PARylation sites from our previous study^16^ as a reference. Peptides derived from these proteins were identified, and only the peptides with signal-to-noise ratio (S/N) values >5 for both heavy and light species were retained.

Next, we calculated the PARylation stoichiometry for each protein based on results obtained from all four mix ratios. Through these experiments, we successfully determined the PARylation stoichiometries of 235 proteins (Figure 1C). Among them, 215 proteins were identified from Replica Set 1, and 228 proteins were obtained from Replica Set 2 (Table S1). A total of 206 proteins overlapped between the two Replica Sets (Figure 1C). The PARylation occupancy measurements for these 206 overlapping proteins exhibited a robust correlation among replicates (Figure 1D).

The median and mean PARylation stoichiometries of these PARylated proteins were 0.58 and 2.14%, respectively (Figure 1E). Merely 11% (26/235) of the proteins exhibited PARylation occupancy greater than 5%, while a remarkable 62% (146/235) of the proteins displayed occupancy levels below 1% (Figure 1F). These findings suggest a low overall protein PARylation occupancy.

Furthermore, we observed that PARylation stoichiometry varies by 3 orders of magnitude among these proteins, ranging from 0.01 to 35.48% (Table S2). The wide range of PARylation stoichiometries is consistent with PARylation being a highly dynamic protein modification that is regulated in a spatiotemporal manner.^41^ This feature again highlights the importance of employing different mix ratios to generate SILAC peptide pairs with suitable dynamic ranges and to broadly capture the extensive variation in PARylation levels. For instance, in the case of the FEN1 protein (PARylation stoichiometry = 0.03%), we were able to detect both the heavy and light species across all four mixing ratios, with the relative intensity of the heavy peak increasing as the mix ratio increased (Table S1). Conversely, for the HNRNPUL1 protein (PARylation stoichiometry = 2.03%), which exhibited a PARylation occupancy 60 times higher than that of FEN1 according to our calculations, the heavy species was undetectable at the lowest mix ratio but became discernible at higher mix ratios as its relative intensity increased (Table S1).

PARP1 emerged as one of the most heavily PARylated proteins, with a PARylation stoichiometry of 32.94% under these conditions (Table S2). Consistently, our measurements revealed the PARylation stoichiometry for several proteins known to undergo PARylation, including PARP1, YY1, CTCF, XRCC1, H2AFX, H2AFY, POLR1A, and POLR2A (Figure 1G, and Table S2).^42–45^

To further assess the reproducibility of our findings, we plotted the median heavy-to-light peak area ratio for various proteins as a function of the mixing ratio. We found that the peak ratios and linearity for the same protein exhibited excellent agreement between the two replicate sets (Figure 1H). Furthermore, we also performed independent biochemical experiments to validate our MS stoichiometry data. We generated lysates from H_2_O_2_-treated shPARG HCT116 cells and isolated PARylated proteins using the boronate beads. The PARylated proteins were then eluted, and their abundances were compared to those in serial dilutions of the crude cell lysates. Our MS measurements indicated a PARylation stoichiometry of 0.63% for PCNA and 2.08% for EWSR1, which were consistent with the results obtained from the immunoblotting analysis (Figure 1I).

To evaluate the physiological relevance of our stoichiometric measurements, we sought to further validate our approach in wild-type cells under similar genotoxic stress conditions. We treated wild-type HCT116 cells with H_2_O_2_ (2 mM, 5 min) and subjected the lysates to boronate affinity enrichment, followed by quantitative immunoblotting. Consistent with our MS-based stoichiometry data, PCNA was detected as a PARylated protein in the boronate-enriched fraction. By comparing the abundance of the boronate-enriched fraction to that in the input, we determined the stoichiometry of PCNA to be 0.48% in these WT cells (Figure 1J). This number was similar to that measured in the shPARG cells, indicating that shPARG cells represent a robust system for PARylation stoichiometry analyses.

### Networks of PARylated Proteins with High PARylation Stoichiometries

To further explore the potential roles of PARylation in DNA damage response, we analyzed the functional connections among the 235 identified PARylated proteins (Figure S1). Our analysis revealed that 71 proteins formed five networks, with functions associated with chromosome organization, regulation of gene expression, DNA repair, RNA metabolic process, and cell cycle (Figure 2A).

**Figure 2:**
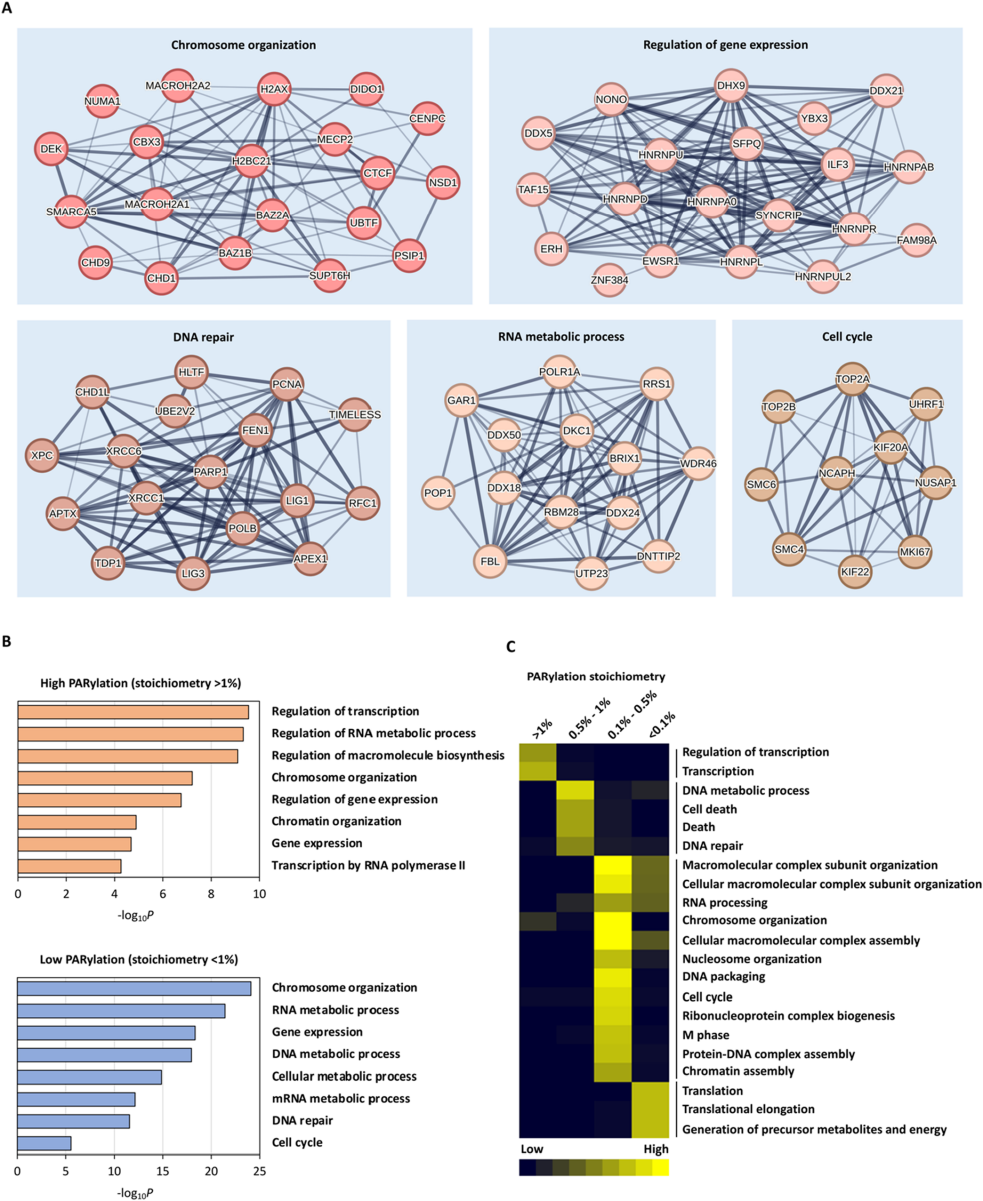
High-occupancy proteins are concentrated in transcription regulators, rather than DNA repair factors. (A) Network presentation of functional connections among the 235 PARylated proteins within the top five largest clusters identified through PARylation stoichiometry analysis. (B) Enriched biological processes in proteins with high (>1%) and low (<1%) PARylation stoichiometry. (C) Clustering of the enriched biological processes across different ranges of PARylation stoichiometry. The previously identified PARylated proteins (Table S13, Zhang et al., *Nature Methods*)^16^ were used as a reference.

To gain further insights into the functional implications of PARylation stoichiometry, we conducted a GO analysis to identify enriched biological processes (BP) across different stoichiometry ranges (Figure 2B,C). The PARylated proteins were categorized into four groups based on their PARylation stoichiometries: >1, 0.5–1%, 0.1–0.5%, and <0.1%. It is worth noting that for the BP enrichment analysis, we employed the list of identified PARylated proteins as the background list, thus ensuring that the enriched processes were specifically associated with different stoichiometry values rather than being influenced by the entire human genome. Subsequently, we examined the enriched biological processes for each stoichiometric category and clustered them accordingly (Figure 2C). Consistently, proteins with the highest occupancy (>1%) exhibited significant enrichment in GO terms related to “transcription and regulation of transcription” (Figure 2C). Processes such as “DNA repair”, “DNA metabolic process”, and “cell cycle” were enriched in proteins with PARylation stoichiometries between 0.5 and 1% (Figure 2C). Our comprehensive analysis of PARylation stoichiometry thus indicates that high-occupancy proteins are predominantly concentrated in transcription regulators.

We further investigated the characteristics of proteins displaying the highest levels of PARylation occupancy (>1%) (Figure 3A and Table S2). Notably, PARP1 emerged as a central hub protein, exhibiting the most extensive functional connections among the 91 highly PARylated proteins (Figure 3A). This aligned with its well-known role as a key mediator of cellular PARylation. The list of proteins with high PARylation stoichiometries also included several well-established PARylated proteins, including YY1, TAF15, CHTOP, GAR1, and TIMELESS.^16,38,46,47^ Again, globally, the highly occupied (>1%) proteins were significantly enriched in transcription and regulation of transcription (Figure 2C). Remarkably, within the top 91 proteins exhibiting the highest occupancy levels (>1%), as many as 57 proteins (62.64%) were identified as transcription regulators (Figure 3B).

**Figure 3:**
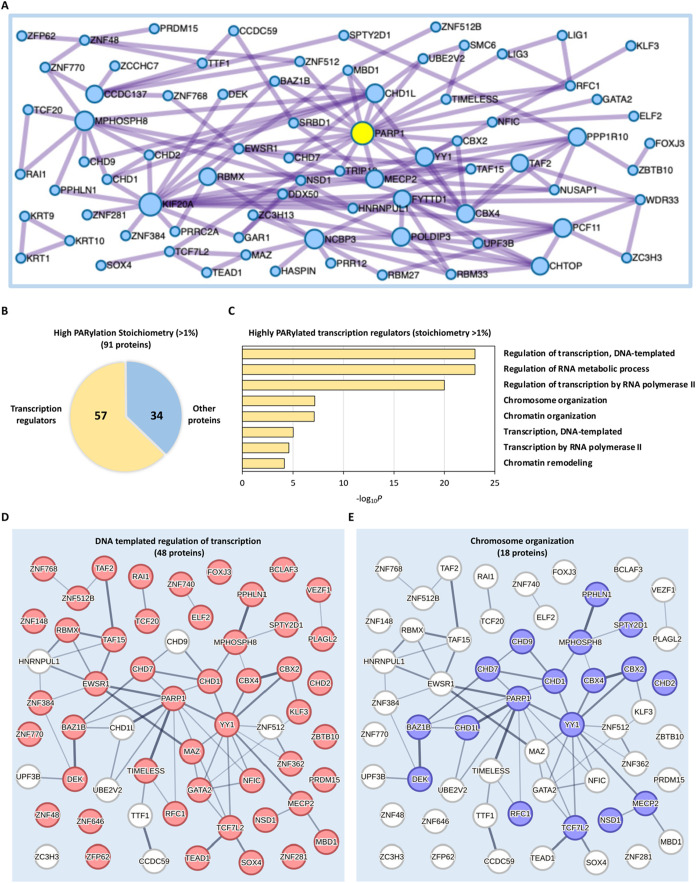
PARylation mainly regulates transcription during the DNA damage response. (A) Protein–protein interaction enrichment analysis of the 91 highly PARylated proteins (stoichiometry >1%). (B) Proportion of transcription regulators among the top 91 highly PARylated proteins (stoichiometry >1%). (C) Enriched biological processes observed among the 57 transcription regulators within the top 91 highly PARylated proteins (stoichiometry >1%). (D, E) Network presentation of functional connections among the 57 highly PARylated transcription regulators within the two largest clusters identified through PARylation stoichiometry analysis. Proteins involved in DNA-templated regulation of transcription are marked in red (D), while proteins that participate in chromosome organization are marked in blue (E).

The pronounced occupancy of PARylation in transcription regulators motivated us to delve deeper into their specific characteristics. To achieve this, we conducted a further GO analysis focusing on these highly PARylated transcription regulators (Figure 3C). Furthermore, we found that these proteins formed two large networks, with functions predominantly associated with DNA-templated regulation of transcription (48 proteins) and chromosome organization (18 proteins), respectively (Figure 3D,E). It has been well established that PARylation plays a central role in facilitating the recruitment of DNA repair factors to DNA lesions.^48^ Our comprehensive PARylation stoichiometry analysis sheds new light on the significance of PARylation in controlling other biological processes in the nucleus, in particular, transcriptional regulation and chromatin remodeling. This perspective expanded the functional repertoire of PARylation beyond DNA repair factor recruitment.

### Transcription Regulators Have High PARylation Stoichiometries

Our large-scale PARylation stoichiometry studies indicated that proteins involved in transcription regulation and chromatin remodeling have high PARylation stoichiometries. We further studied the 48 highly PARylated proteins (PARylation stoichiometries >1%) that are involved in DNA-templated regulation of transcription. We found that more than 60% (29/48) of the proteins in this network were identified as transcription factors (Figure 4A). Remarkably, protein domain analyses demonstrated that the majority (55.17%, 16/29) of these highly PARylated transcription factors are Cys2-His2 zinc finger (C2H2-ZF) proteins (Figure 4B), which represent the largest group of putative human transcription factors (Figure 4C,D). All of these ZNF proteins are C2H2-type zinc finger transcription factors that target specific binding motifs on gene transcription starting sites (Figure 4E,F). In this context, recent studies showed that proteins with C2H2-type zinc finger domains represent a major class of PAR-binding proteins.^49,50^

**Figure 4:**
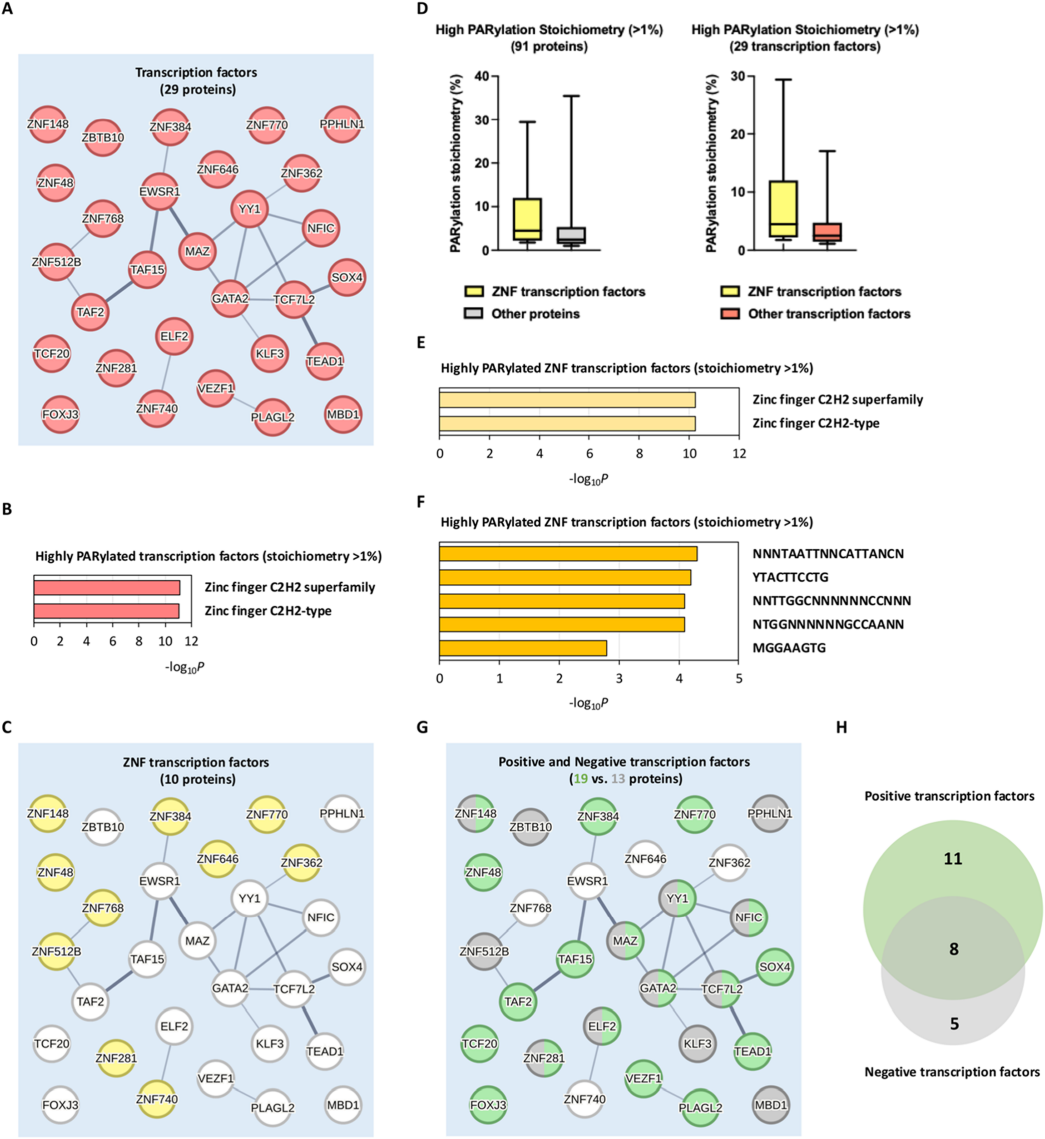
PARylation regulates transcription through modulating transcription factors. (A) Network presentation of functional connections among the 29 transcription factors within the 48 significantly PARylated transcription regulators (stoichiometry >1%) involved in DNA-templated regulation of transcription. (B) Protein domain analysis of the 29 highly PARylated transcription factors (stoichiometry >1%). (C) Network presentation of functional connections among a subset of highly PARylated ZNF transcription factors within the 29 highly PARylated transcription factors (stoichiometry >1%). (D) Distribution of PARylation stoichiometries among the ZNF transcription factors within the 91 highly PARylated proteins (stoichiometry >1%) (left), or within the 29 highly PARylated transcription factors (stoichiometry >1%) (right). The box represents the interquartile range, the middle line denotes the median, and the whiskers indicate the minimum and maximum values excluding outliers. (E) Protein domain analysis of the 10 highly PARylated ZNF transcription factors (stoichiometry >1%). (F) Transcription factor analysis of the 10 highly PARylated ZNF transcription factors (stoichiometry >1%). (G) Network presentation of functional connections among the positive (green) or negative (gray) transcription factors within the 29 highly PARylated transcription factors (stoichiometry >1%). (H) Biological processes analysis identified 19 positive transcription factors and 13 negative transcription factors among the 29 highly PARylated transcription factors (stoichiometry >1%). Eight multifunctional transcription factors overlapped with both positive and negative properties.

These 29 highly PARylated transcription factors contained both positive (19 proteins) and negative (13 proteins) regulators of DNA-templated transcription (Figure 4G,H). Among these proteins, we identified 8 multifunctional transcription factors, including YY1, GATA2, MAZ, NFIC, TCF7L2, ELF2, ZNF281, and ZNF148 (Figure 4G,H). These 8 proteins can exhibit both positive and negative control over a substantial number of cellular genes by binding to their respective transcription start sites.^51–66^

For example, the transcription factor Yin Yang 1 (YY1) is a critical component of the RNA Pol II transcription machinery and is a known PARylated protein (Figure 4A).^42^ As a transcriptional repressor protein, the PARylation of YY1 has been shown to trigger its dissociation from the PARP1 promoter binding site. This release of YY1 alleviates the repression of PARP1 transcription, which represents an important positive-feedforward mechanism to promote PARP1 expression and PARylation activity in response to cellular insults.^52^ In our investigation, we observed that YY1 exhibited a remarkably high PARylation stoichiometry of 17.05% under oxidative DNA damage conditions (Figure 4A and Table S2). It is conceivable that PARylation stoichiometries could be a key factor that regulates the affinity of transcription factors toward their cognate DNA-binding sites, thus mediating the expression of genes critical to DNA damage response.

In addition, TATA-box binding protein-associated factor 15 (TAF15) is a transcription factor that belongs to the FUS, EWS/EWSR1, TAF15 (FET) protein family.^67,68^ The FET family proteins play critical roles in transcriptional regulation, and their dysregulation through chromosomal translocations generates oncogenic fusion proteins that drive malignancies such as sarcomas and leukemias.^69^ These fusion oncoproteins typically retain the N-terminal low-complexity domain (LCD) of FET proteins, which facilitates phase separation and transcriptional condensate formation. At the same time, they incorporate the DNA-binding domain (DBD) of partner transcription factors, enabling aberrant gene regulation.^69,70^ For example, EWSR1–FLI1 is involved in the pathogenesis of Ewing’s sarcoma. It is formed as a result of a chromosomal translocation, which fuses the *EWSR1* gene’s N-terminal transcriptional activation domain with the C-terminal ETS-family DBD of *FLI1*.^71,72^ This chimeric protein acts as a potent transcriptional activator or repressor, depending on target gene context.^71,73^ Mechanistically, EWSR1–FLI1 undergoes phase separation at GGAA-rich loci, forming biomolecular condensates that recruit RNA polymerase II and coactivators, thereby amplifying transcriptional output.^69^ Similarly, a TAF15 fusion protein is a result of a chromosomal rearrangement between the TAF15 gene and another transcription factor gene like *NR4A3* or *ZNF384*.^74,75^ These fusion proteins are found in sarcomas and some other cancers, such as extraskeletal myxoid chondrosarcoma or leukemia. They are considered strong transcription factors that can activate or repress genes.^71–75^

Furthermore, recent studies showed that all three FET proteins form pathological aggregates that are detected in various neurodegenerative diseases.^76–80^ For example, abundant amyloid filaments of TAF15 were recently found in brain samples from frontotemporal dementia (FTD) patients.^76^ The filament fold is formed from residues in the low-complexity domain (LCD) of TAF15, and it is recurrently observed in TAF15 filaments residing in the motor cortex and brainstem of these patients. The formation of TAF15 amyloid filaments with a characteristic fold in FTLD establishes TAF15 proteinopathy in neurodegenerative disease.^76^ In addition, EWSR1 is a prion-like protein, whose LC (low complexity) domains are well-known to form stress-induced biomolecular condensates.^81^ Importantly, *EWSR1* mutations have been identified in patients with familial ALS and FTD.^82^ The ALS-associated mutant EWSR1 proteins exhibit an enhanced propensity for protein aggregation compared to WT EWSR1, suggesting that these mutations may precipitate accelerated aggregation within affected motor neurons.^83^

We previously found that TAF15 and EWSR1 were extensively PARylated with a total of 16 and 8 Asp-/Glu-PARylation sites, respectively.^16^ Furthermore, PARylation of TAF15 and EWSR1 was highly sensitive to the treatment of PARP inhibitors (e.g., Olaparib), suggesting that they are potential PARylation substrates of PARP1.^16^ In this current study, we demonstrated that TAF15 is a highly PARylated protein with a PARylation stoichiometry of 7.28% under oxidative DNA damage conditions (Figure 4A and Table S2). We also validated these results in WT-HCT116 cells using boronate affinity enrichment followed by quantitative immunoblotting. From these experiments, we found that TAF15 was PARylated with a stoichiometry of 3.10% in WT cells (Figure 1J). Furthermore, we found that EWSR1 (EWS) also exhibited high levels of PARylation (stoichiometry of 2.08%) under genotoxic stress conditions (Figure 4A and Table S2). Importantly, it is increasingly appreciated that PARylation is a critical seeding mechanism that promotes the phase separation and aggregation of many intrinsically disordered proteins, including the FET proteins.^84,85^ It is conceivable that PARylation stoichiometry may serve as a critical determinant governing the spatiotemporal dynamics of these FET proteins in the context of transcriptional regulation and biomolecular condensation.

### Chromatin Remodelers Have High PARylation Stoichiometries

Our functional network analysis showed that a large number of highly PARylated transcription regulators are interconnected between DNA-templated regulation of transcription and chromosome organization (Figures 3E and 5A). Notably, a majority (16 of 18) of the highly PARylated chromatin remodelers directly participate in DNA-templated transcriptional regulation (Figure 5B). This suggests that PARylation exerts its regulatory influence on multiple facets of the transcriptional processes through diverse mechanisms, including the modulation of chromatin remodeling.

**Figure 5:**
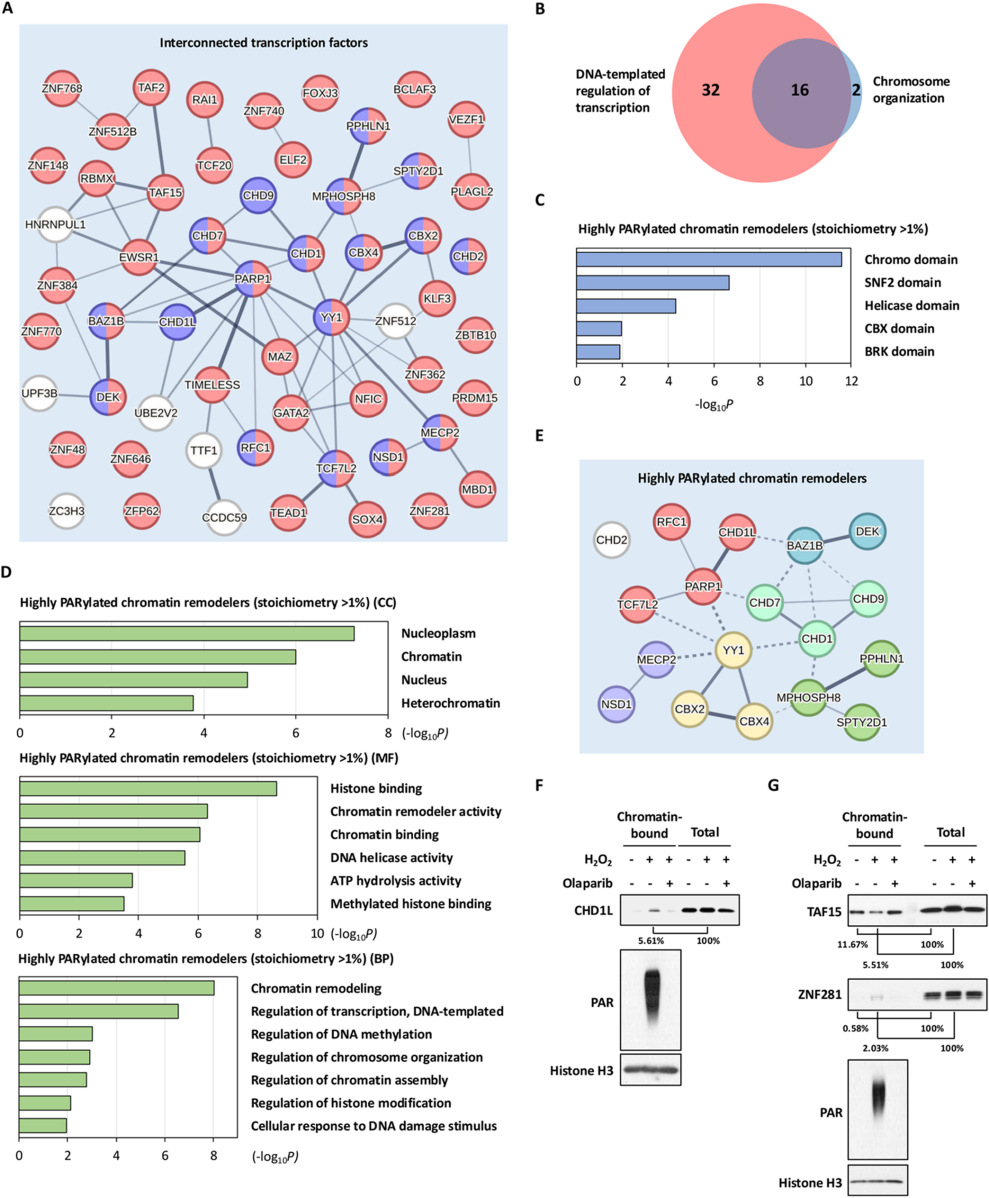
PARylation modulates transcription through chromatin remodeling. (A) Functional interconnection network of the highly PARylated transcription regulators (stoichiometry >1%) involved in DNA-templated regulation of transcription and chromosome organization. (B) Functional connection network analysis identified 16 highly PARylated transcription factors (stoichiometry >1%) involved in both DNA-templated regulation of transcription and chromosome organization. (C) Protein domain analysis of the 18 highly PARylated chromatin remodelers (stoichiometry >1%). (D) GO analysis of the 18 highly PARylated chromatin remodelers (stoichiometry >1%). CC, cellular component; MF, molecular function; BP, biological process. (E) Functional clustering networks of the 18 highly PARylated chromatin remodelers (stoichiometry >1%). (F) PARylated forms of CHD1L are enriched on chromatin. Cells treated with H_2_O_2_ (2 mM, 5 min) and/or Olaparib (1 μM, 40 min) were lysed. Lysates were subject to chromatin fractionation. Input lysates (Total) and proteins in the chromatin-bound fraction were analyzed by immunoblotting with antibodies against CHD1L, PAR, and Histone H3, respectively. For CHD1L, quantification of the chromatin-associated fraction compared to its global level in whole-cell lysates is shown. (G) Validation of stoichiometry-linked PARylation-dependent chromatin relocalization for TAF15 and ZNF281. Cells treated with H_2_O_2_ (2 mM, 5 min) and/or Olaparib (1 μM, 40 min) were lysed. Lysates were subject to chromatin fractionation to isolate chromatin-bound proteins. Input lysates (Total) and chromatin-bound fractions were analyzed by immunoblotting with antibodies against TAF15, ZNF281, PAR, and Histone H3, respectively. Quantification of chromatin-associated TAF15 and ZNF281 compared to their global level in whole-cell lysates is shown.

We conducted a protein domain analysis of the 18 highly PARylated transcription regulators involved in chromosome organization (Figure 3E). Our analysis revealed a significant enrichment of the Chromatin organization modifier domain (Chromodomain), Helicase domain, and SNF2 domain (ATPase domain) within these proteins (Figure 5C). These findings align with the established functions of these proteins in chromatin remodeling.^86–88^ Furthermore, GO analysis indicated that these highly PARylated substrates predominantly consisted of chromatin-associated proteins with chromatin remodeler activity and helicase activity involved in chromatin remodeling and DNA-templated regulation of transcription (Figure 5D). Importantly, our functional network analysis indicated that these highly PARylated chromatin remodelers regulate various aspects of chromatin organization through diverse mechanisms such as the regulation of chromatin accessibility, DNA methylation, and gene expression (Figure 5E). Overall, these observations suggest that PARylation may regulate transcription through the modulation of chromatin remodeling processes.

Chromatin remodelers have recently been recognized as important signaling coordinators in DNA damage response.^89^ Compelling evidence indicates that PARylation governs access and activities of certain chromatin remodelers, as exemplified by MECP2, CHD1L (ALC1), and DEK.^86–88^ For instance, MECP2 is a chromosomal protein that promotes chromatin clustering. However, PARylation of MECP2 has a negative effect on higher-order chromatin structure, leading to chromatin decompaction.^86^ We identified MECP2 as a highly PARylated protein (stoichiometry of 2.23%) in response to genotoxic stress treatments (Figure 5E and Table S2).

PARylation is known to mediate the recruitment of different chromatin remodeling enzymes to the DNA damage site via their PAR-binding domains.^90–92^ In this context, certain chromatin remodeling complexes can induce chromatin relaxation, enhancing the DNA accessibility for transcriptional activation. One such example is the Chromodomain-Helicase-DNA-binding protein (CHD) 1-like (CHD1L, also known as ALC1), an SNF2-like ATPase that is recruited to DNA lesions by PAR through its macrodomain motif. This then triggers chromatin relaxation,^87^ a process that is essential for effective transcriptional control.^93^ Furthermore, PAR binding could also mediate the recruitment of repressive chromatin modifiers, where they bind to PAR and exert inhibitory effects on transcription. For example, the human oncoprotein DEK, a chromatin architectural protein, maintains a heterochromatic state in Drosophila through its interaction with PAR via its PBM domain.^88^ Notably, we observed robust PARylation of both CHD1L (stoichiometry of 5.67%) and DEK (stoichiometry of 1.67%) in response to genotoxic stress treatments. These results suggest that direct PARylation of these proteins may also contribute to the functional regulation of their activities (Figure 5E and Table S2). The presence of PAR moieties on these chromatin remodelers could also sterically disrupt protein–protein interactions (PPIs) and impede the formation of certain protein complexes during the chromatin remodeling process.

Collectively, our PARylation stoichiometry analysis provides compelling evidence that PARylation serves as a coordinator of multiple facets within the transcriptional regulation processes, employing diverse mechanisms, such as the regulation of chromatin remodeling and the coregulation of transcription factors in response to genotoxic stress. These findings highlight the multifaceted role of PARylation in orchestrating the intricate network of regulatory events involved in maintaining the genomic integrity and gene expression.

### Functional Studies of Transcription Factors and Chromatin Remodelers with High PARylation Stoichiometries

To directly test whether PARylation stoichiometry influences the chromatin-associated function of high-occupancy substrates, we performed chromatin fractionation assays coupled to quantitative immunoblotting. We focused on a representative chromatin remodeler, CHD1L, which has a high PARylation stoichiometry of 5.67%, as measured in our global PARylation stoichiometry experiments. In H_2_O_2_-treated cells, we isolated chromatin-bound proteins and measured the level of CHD1L within this compartment. We observed clear PARylation-dependent recruitment of CHD1L to chromatin (Figure 5F). Specifically, we found that CHD1L was markedly enriched on chromatin after DNA damage, and this effect was completely blocked by pretreating the cells with a PARPi (i.e., Olaparib) (Figure 5F). Remarkably, when normalized to its total cellular levels, the calculated chromatin-associated fraction of CHD1L (under genotoxic conditions) is 5.61% of the total amount of CHD1L in whole-cell lysates (Figure 5F). This number is very close to the PARylation stoichiometry of CHD1L (5.67%), providing direct evidence that high PARylation stoichiometry correlates with a specific functional state (e.g., chromatin association). The elevated PARylation occupancy on chromatin suggests that PARylation may positively regulate the recruitment or retention of these remodelers to genomic loci upon DNA damage. CHD1L is a known PAR-dependent chromatin remodeler that is recruited to the chromatin via its PAR-binding macrodomain. Furthermore, our previous Asp-/Glu-PARylation proteomic studies showed that CHD1L is a highly PARylated protein with 10 identified Asp-/Glu-PARylation sites.^16^ These results suggest that covalent PARylation could directly modulate its ATPase activity and protein interactions. The results from our current experiments bridge our stoichiometric measurements with a specific functional outcome, demonstrating that the PARylation occupancy is a key parameter governing the chromatin-regulatory functions of these proteins.

Building upon the functional data for CHD1L, we sought to further assess the functional implications of other proteins with high PARylation occupancy. We previously performed a chromatin relocalization screen in which we identified proteins that were either enriched (defined as “chromatin-ON”) or depleted (defined as “chromatin-OFF”) from the chromatin, upon PARylation induction.^94^ We cross-referenced the proteins from this screen to those identified from the current PARylation stoichiometry study. Interestingly, this comparison revealed a compelling correlation: proteins exhibiting high PARylation stoichiometries often displayed corresponding PAR-dependent chromatin relocalization regulation in the earlier screen.^94^ For instance, TAF15, identified here with a high PARylation stoichiometry (7.28%), was previously categorized as a “chromatin-OFF” target whose dissociation from chromatin upon DNA damage was PARylation-dependent. Conversely, ZNF281 was identified to be a highly PARylated transcription factor with a PARylation stoichiometry of 2.35%. This protein was classified as a “chromatin-ON” protein, showing PAR-dependent recruitment to chromatin.

To experimentally bridge these data sets, we performed chromatin fractionation assays for TAF15 and ZNF281 under PARP-active (i.e., H_2_O_2_-treated) and PARP-inhibited (i.e., Olaparib-treated) conditions (Figure 5G). Consistent with our previous study,^94^ H_2_O_2_ treatment induced a substantial decrease in chromatin-associated TAF15 (chromatin-OFF regulation). These effects were reversed by the Olaparib treatment. Importantly, the percentage of TAF15 being depleted from chromatin was closed correlated with its PARylation stoichiometry values. On the other hand, PARylation promoted the chromatin association of ZNF281 (chromatin-ON regulation). This congruence between quantitative PARylation occupancy and PAR-dependent chromatin relocalization reinforces the biological relevance of our stoichiometric measurements. It demonstrates that for a PARylated protein, its high PARylation levels could be functionally linked to the dynamic, PAR-mediated regulation of substrate localization, particularly for transcription regulators and chromatin remodelers, during DNA damage response.

## Discussion

Stoichiometry (the fraction of a given protein that is modified at a given time) is a critically important parameter when assessing the functional importance of a protein post-translational modification event. Wu et al.^95^ previously developed an elegant strategy to globally measure protein phosphorylation stoichiometries. They utilized phosphatase treatment combined with LC-MS/MS analysis to determine proteome-wide phosphorylation stoichiometry.^95^ Peptides resulting from whole-cell lysate digestion were split in half. One fraction was treated with phosphatase, and the other was not. The two fractions were chemically labeled with heavy and light tags, respectively, mixed, and analyzed by LC-MS/MS. The difference between the heavy and light species of a peptide containing known modification sites thus represents the phosphorylation stoichiometries. As another example, Weinert et al. presented a strategy for quantifying global acetylation stoichiometries.^96^ Due to the very low stoichiometry of acetylation, direct comparison between enriched acetylated peptides and nonacetylated forms was not feasible. Instead, an in vitro chemical acetylation labeling method was employed, revealing that 86% of the sites were acetylated at less than 1% occupancy.

However, characterization of the stoichiometries of many other PTMs has been challenging.^97^ This is particularly the case for PARylation. First, PARylation is a polymeric or heterogeneous modification that lacks a defined mass shift. As a result, direct detection of the PARylated species (and then comparison of the ratio of the PARylated/unPARylated species to determine the PARylation stoichiometries) using MS-based methods is not feasible. Second, PARylation is of low abundance, and a robust enrichment strategy needs to be developed for its stoichiometric analyses. Finally, compared to other common PTMs, a rather unique feature of PARylation is that PARylation can occur on various amino acid acceptors, including Asp, Glu, Lys, Arg, Ser, Cys, His, and Tyr.^48,98^ It is important to note that these PAR-amino acid linkages display drastically different stability.^16,31,35,36,99^

We previously developed an integrated strategy toward the site-specific analysis of the cellular Asp-/Glu-PARylated proteome.^16^ These studies have led to the identification of thousands of unique, unambiguously assigned Asp/Glu-ADP-ribosylation sites.^16,100^ Here, we leveraged this method and developed a large-scale method to measure the stoichiometries of protein PARylation. As previously shown, because Asp-/Glu-PARylation is unstable, we used a mild lysis buffer (e.g., a neutral SDS buffer) to deactivate the PARP/PARG enzymes and to universally preserve the PAR chains on all amino acid linkages. Importantly, this buffer system is compatible with the subsequent boronate enrichment procedures. In this study, we further evaluated the boronate enrichment strategy. We found that it had extraordinary efficiency, which allowed for the complete recovery of the PARylated proteins from crude cell lysates. Because the enrichment is based on the formation of chemical bonds between boronate and the 1,2-cis-diol in ADP-ribose, the method enables the enrichment of PARylated proteins, regardless of their amino acid linkages. Finally, compared to protein-based enrichment strategies (e.g., Af1521), the boronate beads are uniquely compatible with subsequent sample preparation procedures, e.g., on-bead proteolytic digestion.

These key features of the boronate beads formed the foundation of our PARylation stoichiometry approach (Figure 1B). Specifically, we used the SILAC approach and generated two samples, i.e., the light sample (with activated PARP1) and the heavy sample (with inhibited PARP1). The PARylated version of a protein in the light sample was recovered using boronate enrichment and then digested. At the same time, whole-cell lysates of the heavy sample were digested to generate the peptides corresponding to the unPARylated version of this protein. By mixing the light peptides and heavy peptides, we were able to determine the PARylation stoichiometries on a global scale. Because of the complete recovery of the PARylated proteins by the boronate affinity beads, this approach allows us to encompass all PARylation events occurring on diverse amino acid side chains.

The Orbitrap mass detector offers a dynamic range of approximately 5000.^40^ However, it is important to note that proteins with very low or high PARylation stoichiometries could generate light or heavy peptides with extreme ratios that might fall outside the optimal dynamic range of the Orbitrap detector. To address this issue, we conducted a titration experiment, generating samples with varying heavy-to-light mix ratios (i.e., 1:50, 1:200, 1:800, and 1:2000). This approach ensured that SILAC peptide pairs with extreme light/heavy ratios under one SILAC mixture sample were recovered in subsequent samples within the titration series.

To investigate the influence of PARylation on protein function and regulation, we conducted an analysis of the enrichment of Gene Ontology categories across different stoichiometry ranges: high (>1%), medium (0.5–1%), low (0.1–0.5%), and very low (<0.1%). Clustering of biological processes based on PARylation stoichiometry values revealed several intriguing findings.

First, the majority of PARylation events occurred at very low levels. At the same time, PARylation stoichiometries also exhibit a wide dynamic range. This is consistent with the synthesis of PAR polymers being highly regulated in a spatiotemporal manner. Upon binding to nicked DNA, PARP1 is rapidly activated, leading to the generation of many PARylated proteins at the DNA lesions.^31^ It has been estimated that the amount of PAR in a cell increases from ∼3000 PAR molecules/cell under basal conditions to >1,50,000 PAR molecules/cell under genotoxic conditions.^101^

Second, among all of the measured proteins, PARP1 demonstrated one of the highest PARylation stoichiometric values (32.94%). This is consistent with the notion that a large fraction of the cellular PAR chains is attached to PARP1 itself.^102^ Protein-linked PAR polymers (PAR chains) serve as a platform for the recruitment of DNA damage proteins (e.g., XRCC1, LIG3, and DNA Polβ) via their PAR-binding domains, which further promote the formation of a large protein complex that mediates the repair of DNA breaks. Because PARP1 is a very abundant nuclear protein and is PARylated with high stoichiometric values, it is expected that PARylated PARP1 plays a central role in acting as a scaffold to orchestrate the recruitment and coordination of DNA damage repair factors through its PAR chains.

Third, PARP1 activity influences several key DNA repair pathways, including base excision repair (BER), single-strand break repair (SSBR), nucleotide excision repair (NER), and nonhomologous end-joining (NHEJ).^103,104^ In our data set, we were also able to determine the PARylation stoichiometry of several proteins associated with these repair machinery (e.g., XRCC1, LIG3, PCNA, XPC, and XRCC6) (Figure 2A). These results suggest that, besides PAR-binding, direct PARylation of these DNA repair factors could also facilitate DNA repair processes. However, we also observed that many of these DNA damage repair factors are associated with low PARylation stoichiometries (Table S2). These results suggest that there could be a complex interplay between PAR binding vs direct PARylation for these DNA repair proteins (Figure 2A). Therefore, whether PARylation regulates the activity of these DNA repair proteins via protein–protein interactions vs covalent PARylation will be examined in future studies.

Fourth, many proteins with the highest stoichiometries were associated with the biological processes of “Regulation of Transcription”. This observation suggests that a significant fraction of these transcription regulator molecules is in a pool that is accessible to PARP1, which becomes PARylated under genotoxic conditions. PARP1 is known to act as a positive cofactor in transcription. At the same time, its activation can also repress RNA polymerase II-dependent transcription.^105,106^ Previous studies have demonstrated that PARP1-mediated transcriptional silencing involves the PARylation of the TATA-binding protein.^106^ Notably, this modification effectively prevents the formation of active transcription complexes (e.g., for the transcription factor YY1 ^52^). However, once transcription complexes are assembled, the initiation of PARylation no longer affects the DNA binding of the transcription factors. Consequently, if transcription factors are already bound to DNA, they become inaccessible to PARylation.^107^ Hence, PARylation inhibits the binding of transcription factors to DNA, while DNA binding prevents their modification. It is plausible to propose that PARylation serves as a molecular switch to block DNA binding of transcription factors, thereby preventing the expression of damaged genes. In our current study, we identified eight highly PARylated multifunctional transcription factors, exhibiting significant PARylation stoichiometries under the oxidative DNA damage conditions (Figure 4H,I and Table S2).

Finally, proteins with high PARylation stoichiometries were also enriched with chromatin remodelers. It is plausible that PARylation reconfigures the chromatin structure, resulting in altered chromatin accessibility and the exposure of DNA, which provides accessible binding sites for transcription factors and DNA repair proteins. Our stoichiometric data set prompted the hypothesis that high PARylation occupancy directly regulates substrate function. To test this for chromatin remodelers, we analyzed a high-stoichiometry protein, CHD1L. We found that it became markedly enriched on chromatin after PARylation induction, with chromatin-associated fractions consistent with its PARylation stoichiometries (Figure 5F). This indicates that high PARylation occupancy correlates with a functionally relevant chromatin-bound state. These functional experiments directly exemplify how our stoichiometry measurements can inform testable hypotheses about PARylation-dependent regulation.

PARylation has garnered considerable attention, particularly due to the tremendous success of PARP inhibitors in the clinic.^108^ Previously, it was summarized that PARylation regulates the first wave of the DNA damage response.^48^ Understanding the stoichiometry of PARylation represents an important parameter in evaluating the impact of PARylation on protein function. We highlighted several protein classes with high PARylation stoichiometries. Among the highly PARylated transcription regulators is a class of poorly studied ZNF proteins. Our analysis revealed substantial PARylation occupancy in these ZNF proteins. We performed cross-reference analyses between the PARylation stoichiometry data set with the results from our previously published chromatin relocalization screen.^94^ These analyses identified a ZNF protein (i.e., ZNF218) that showed an enhanced chromatin association upon the induction of PARylation. Importantly, independent fractionation assays showed that the fraction of chromatin-bound ZNF218 closely correlated with its PARylation stoichiometry. This finding implies that the direct PARylation of these proteins may play a crucial role in regulating their functional properties. Interestingly, a recent study showed that many of these ZNF proteins also bind to PAR polymers.^49,109^ Whether the PARylation of these transcription factors regulates their function positively or negatively, and how specific types of transcription factors are involved in DNA damage response-coupled transcriptional regulation, remains to be elucidated.

In addition, we demonstrated that TAF15 and EWSR1, two members of the FET family of proteins, were extensively PARylated with high stoichiometric values. The FET proteins are DNA/RNA-binding proteins that are implicated in the DNA damage response and transcription regulation. When fused with another gene (FLI1, ATF1, CREM, RBFOX2, ERG, TCF7L2, TFEB, and ZNF384), they form abnormal chimeric transcription factors (e.g., EWSR1-FLI1, EWSR1-ATF1, and TAF15-ZNF384) that drive the pathogenesis of a variety of soft tissue tumors, leukemias, and mesotheliomas.^74,110,111^ These results point to the intriguing possibility of the regulatory importance of PARylation in modulating DNA damage response through transcriptional regulation.

Recent studies showed that the FET proteins also form pathological aggregates that drive the pathogenesis of various neurodegenerative diseases.^76–80^ Indeed, mutations of the FET proteins are identified in patients with familial ALS and FTD, and these mutations greatly accelerate the formation of fibrils.^78,112,113^ Abundant amyloid filaments of TAF15 were recently found in brain samples from FTD patients.^76^ It has been shown that the low complexity domain in these FET proteins functions as prion proteins that drive the formation of biomolecular condensates. Furthermore, our previous PARylation proteomic studies identified a total of 16 and 8 PARylation sites on TAF15 and EWSR1, respectively. Many of the sites on these FET proteins were exquisitely sensitive to PARP inhibitor treatment, indicating that they are novel PARP1 downstream targets.^16^ Interestingly, we showed that a substantial fraction of the TAF15 protein is associated with the chromatin under basal conditions. Upon induction of DNA damage, TAF15 was depleted, in a PARylation-dependent manner, from the chromatin. These results indicate that PARylation represents an important mechanism to regulate their cellular localization.

The RRM (RNA Recognition Motif) in these proteins is known to bind to PAR polymers.^114^ Because these proteins are PARylated with high PARylation stoichiometries, the high local concentrations of PAR polymers in combination with the PAR-binding domains in these proteins could function as a particularly important seeding mechanism (i.e., via multivalency) to promote their chromatin depletion and aberrant biomolecular condensation. This important aspect of PAR biology in the context of neurodegenerative diseases warrants future studies.

Our study offers a large-scale method to measure PARylation stoichiometries. At the same time, it is also important to acknowledge its limitations. Because we used boronate beads to pull down the PARylated proteins, this method allows for the determination of PARylation stoichiometries at the protein level but not specific PARylation sites. Future methodologies are needed to assess the stoichiometries at the level of individual PARylated residues. Several promising avenues can be envisioned to bridge this gap. For example, the development of engineered enzymes, e.g., engineered versions of snake venom phosphodiesterase I or specific bacterial glycohydrolases, capable of cleaving within the PAR polymer to leave a defined, site-attached remnant (such as a phosphoribosyl moiety) would be transformative.^23,115^ Coupling such enzymatic tools with chemically synthesized internal peptide standards (with isotopic labeling) could enable site-specific quantification by targeted mass spectrometric methods (e.g., multiple reaction monitoring). In this context, both boronate affinity enrichment and ongoing advances in chemoenzymatic tagging of ADP-ribose could provide robust handles for the site-specific isolation of PARylated peptides. Nevertheless, we highlight that our current protein-level stoichiometry data set provides a valuable and directly integrable framework (Table S1). It can be effectively combined with existing or future site-mapping studies.^16^ For proteins identified with a single dominant PARylation site, the protein-level stoichiometry we report here provides a close approximation of the site-specific value. Conversely, for multisite PARylated proteins, our data define the total pool of the modified protein, which forms an essential context for interpreting the relative abundance and functional contribution of modifications at individual residues. Therefore, we believe that the convergence of the foundational protein-level quantification established here with these evolving site-resolution techniques will collectively propel the field toward the precise residue-resolution mapping of PARylation stoichiometry. Achieving this level of precision is critical for functional decoding of this complex and dynamic modification.

Our quantitative measurements were obtained under defined conditions using PARG-deficient cells, a system optimized to capture a broad and stable PARylome for methodological development. Consequently, the absolute stoichiometry values presented herein represent a specific “snapshot” of this undoubtedly dynamic protein modification. They are likely influenced by variables, including the type and intensity of genotoxic insult, the cellular PARP/PARG activity balance, cell cycle stage, and tissue type. Nonetheless, our central biological finding, the preferential high PARylation occupancy of transcription regulators, was validated in wild-type cells. While absolute occupancy levels may vary depending on the specific cellular contexts, the relative functional ranking of the protein classes is expected to be conserved. Therefore, this data set serves as a foundational quantitative resource and a critical benchmark. It will enable future comparative studies to systematically quantify how PARylation occupancy remodels across diverse physiological and pathological contexts, from development to disease.

Shifting from method development to application, an equally important question is the stoichiometry pipeline’s adaptability to clinical samples, primary tissues, or different model systems. Translating this approach presents specific challenges yet is strategically enabled by its core design. Key practical hurdles must be acknowledged, including the requirement for substantial input material from often limited biopsies, the critical need for immediate sample stabilization to preserve the native PARylation state, and the interpretative complexity arising from tissue heterogeneity. Nevertheless, the inherent feasibility of this translation is underpinned by our method’s strategic advantages: the exceptional enrichment efficiency of boronate chemistry is ideal for low-abundance targets in complex lysates, and the SILAC-based framework is readily adaptable via heavy-labeled spike-in standards for precise quantification in unlabeled specimens. Indeed, this is facilitated by the fact that a “heavy” cell lysate could be used as a common internal standard that is mixed with different clinical samples for relative quantification. These considerations chart a clear future pathway; by positioning our cell-line data set as a quantitative benchmark and our protocol as a versatile platform, we directly enable future studies to profile PARylation occupancy in patient cohorts or primary models, thereby transitioning the tool from basic discovery to translational research.

Here, we describe a novel, large-scale approach for the measurement of PARylation stoichiometries. Because PARylation is a labile, heterogeneous, and low-abundance modification, we performed extensive optimization of our approach, including cell lysis conditions, boronate enrichment efficiency, and carefully designed titration experiments. In total, we were able to measure the PARylation stoichiometries of 235 proteins under genotoxic conditions. Our analysis revealed that protein PARylation stoichiometry varies significantly among PARylated proteins, with pronounced dependence on specific biological processes. Functional analyses of this data set revealed a remarkable breadth of regulatory events associated with the PARylated proteins, including DNA damage repair but also RNA metabolism, transcriptional regulation, and chromatin remodeling. We unveiled that proteins with high PARylation stoichiometries are involved in coordinating various aspects of transcriptional regulation and chromatin remodeling during the DNA damage response. The quantitative proteomics approach and the associated data sets described herein represent a valuable resource for system-level investigations into the functions of protein PARylation dynamics.

## Materials and Methods

### Cell Culture and Reagents

HCT116 cells with PARG knockdown were generated following previously described methods.^16^ The HCT116 shPARG cells were cultured in a SILAC medium. SILAC Dulbecco’s Minimal Essential Medium (DMEM) was supplemented with 10% dialyzed FBS and 2 μg/mL puromycin. To prepare the SILAC media, 84 μg/mL light Arginine was added to the medium, and 152 μg/mL light or heavy Lysine was added to prepare the light or heavy DMEM media. Anti-poly­(ADP-ribose) antibody was obtained from Tulip Biolabs (#1020). PCNA (#2586), PARP1 (#9542), and GAPDH (#5174) antibodies were purchased from Cell Signaling Technology. EWSR1 antibody (#A300–417A) was purchased from Bethyl Laboratories. *M-aminophenyl-boronic* acid–agarose (A8312) was obtained from Sigma. Unless specified, all other chemicals and reagents were obtained from Sigma.

### Boronate Affinity Beads (BAB) Pulldown

The purification of PARylated proteins for immunoblotting analysis was conducted following the previously described method.^16^ In brief, H_2_O_2_-stimulated cells were lysed using SDS lysis buffer (1% SDS, 10 mM HEPES, pH 7.0, 2 mM MgCl_2_, and 250 U universal nuclease). The cell lysates were then subject to BAB enrichment at RT for 1 h. Subsequently, fresh BAB was added to the mixture and rotated for an additional hour. Crude lysates, as well as the supernatants from the first and second rounds of adsorption, were collected and subjected to immunoblotting analysis to demonstrate the quantitative adsorption of PARylated proteins. Immunoblotting was also used to validate our stoichiometry measurement. The PAR modified proteins were eluted from the BAB using 3 M NH_4_Ac, pH 5.0, and the eluted protein solution was diluted with SDS lysis buffer to the same volume as the starting cell lysates. Meanwhile, a fraction of the cell lysates was diluted to 1/25–1/200 of the original concentration using SDS lysis buffer. Subsequently, equal volumes of the serially diluted cell lysates (input) and the pull-down eluates were analyzed by immunoblotting.

### Immunoblotting Analysis

The samples were subjected to electrophoresis by using the standard SDS-PAGE method. Subsequently, the proteins were transferred onto a nitrocellulose membrane (Whatman). The membranes were then blocked with a TBST buffer (25 mM Tris-HCl, pH 7.5, 150 mM NaCl, and 3% BSA) and incubated overnight at 4 °C with primary antibodies. Following this, peroxidase-conjugated secondary antibodies were applied for 1 h at RT. The blots were developed by using enhanced chemiluminescence, exposed on autoradiograph films, and processed using standard methods.

### Cellular Fractionation

Following procedures previously described,^116^ cells were fractioned using a subcellular protein fractionation kit (Thermo Fisher Scientific) according to the manufacturer’s instructions. Briefly, cells were harvested with trypsin-EDTA, centrifuged at 500*g* for 5 min, and washed with ice-cold phosphate-buffered saline (PBS). After the addition of cytoplasmic extraction buffer (CEB) to the cell pellet, the tube was incubated at 4 °C for 10 min with gentle mixing. Following centrifugation at 500*g* for 5 min, the supernatant (cytoplasmic extract) was transferred to a clean prechilled tube on ice. Next, the MEB buffer was added to the pellet. The tube was briefly vortexed and was incubated at 4 °C for 10 min with gentle mixing. The tube was then centrifuged at 3000*g* for 5 min, and the supernatant (membrane extract) was transferred to a clean prechilled tube on ice. An ice-cold nuclear extraction buffer (NEB) was added to the pellet, and the tube was vortexed at the highest setting for 15 s. Following incubation at 4 °C for 30 min with gentle mixing, the tube was centrifuged at 5000*g* for 5 min, and the supernatant (soluble nuclear extract) was transferred to a clean prechilled tube on ice. Last, an RT NEB buffer containing micrococcal nuclease and CaCl_2_ was added to the pellet. The tube was vortexed for 15 s and was incubated at RT for 15 min. After incubation, the tube was centrifuged at 16,000*g* for 5 min and the supernatant (chromatin-bound nuclear extract) was transferred to a clean prechilled tube on ice.

### Sample Preparation for Stoichiometry Measurement by MS

Light Lys and heavy Lys-labeled shPARG HCT116 cells were treated with H_2_O_2_ (2 mM, 5 min) or Olaparib (1 μM, 40 min), respectively. Both light and heavy cells were lysed using SDS lysis buffer. The protein was quantified using a BCA assay kit, followed by reduction, alkylation, and chloroform/methanol precipitation. The heavy lysate protein was resuspended in urea (pH 7.5) to a final concentration of 250 μg/mL and digested overnight with Lys-C at a 1:100 enzyme-to-protein ratio. Simultaneously, approximately 65 mg of light lysate protein was enriched for PARylated proteins using BAB according to the previously described method section. The protein-bound BAB beads were extensively washed using SDS wash buffer (1% SDS, 100 mM HEPES, pH 8.5, 150 mM NaCl), followed by 100 mM HEPES (pH 8.5) and 150 mM NaCl to remove any residual detergent. Finally, the beads were resuspended in 200 mM HEPES, pH 8.5, and the proteins were on-bead digested overnight with Lys-C. After digestion, aliquots of light and heavy peptides were mixed to different ratios of crude lysate protein from which the peptides are derived (Table 1).

**Table 1:** Sample Preparation

heavy:light mix ratio	heavy lysate protein (μg)	light lysate protein (mg)
1:2000	5	10
1:800	12.5	10
1:200	100	20
1:50	200	10

Depending on the peptide abundance, the mixtures were either directly analyzed using LC-MS/MS or further fractionated using Basic pH RP-HPLC prior to LC-MS/MS analysis. For the samples with ratios of 1:2000 and 1:800, the peptide mixtures were desalted and directly subjected to LC-MS/MS analysis. However, for the samples with ratios of 1:200 and 1:50, the peptide mixtures were desalted and further fractionated using basic pH RP-HPLC (ZORBAX 300Extend-C18, 3.5 μ).

A gradient elution was performed over 90 min, starting from 95% buffer A (10 mM ammonium formate, pH 10.0) to 90% buffer B (10 mM ammonium formate, 90% ACN, pH 10.0). Fractions were collected every 1.5 min from 5 to 80 min, and subsequently concatenated into 17 final fractions based on the scheme presented by Wang et al.^117^ These fractions were then desalted and subjected to LC-MS/MS analysis.

To quantitatively evaluate the nonspecific binding of the non-PARylated form of proteins to the BAB, we employed SILAC-labeled HCT116 shPARG cells. The cells labeled with heavy Lysine were treated with Olaparib (1 μM, 40 min), while the cells labeled with light Lysine were treated with H_2_O_2_ (2 mM, 5 min). Subsequently, 5 mg of heavy cell lysates and 5 mg of light cell lysates were combined and subjected to boronate beads enrichment, followed by on-beads digestion. The resulting mixture of peptides was desalted and analyzed by MS. In this case, the heavy peak represents the nonspecific binder, and the ratio of the heavy peak to the light peak of peptides derived from a PARylated protein was utilized to quantify the extent of nonspecific binding.

### Mass Spectrometry Analysis and Data Processing

Peptide quantification was carried out following the established protocol described previously.^16^ The peptide list was filtered based on the following criteria: (1) the signal-to-noise ratio of either heavy or light peak must be higher than 5; (2) the peptide must be from known PARylated proteins (i.e., present in Table S13, Zhang et al.^16^); and (3) peptides containing known PARylation sites must be excluded. Median of the ratios of heavy peak area to light peak area was subsequently calculated for peptides derived from the same protein.

PARylationstoichiometryatamixratio=(mixratio)/(heavy/lightMSpeakarearatiomedian)

The median of stoichiometries obtained from all of the mix ratios was taken as the final PARylation stoichiometry of the protein.

### GO Pathway Enrichment-Based Clustering of Proteins

The proteins were categorized into four groups based on their PARylation stoichiometry: <0.1%, 0.1–0.5%, 0.5–1%, and >1%. For each group, enrichment analysis of the GO^118^ Biological Processes was conducted separately using DAVID software,^119^ with the entire list of PARylated proteins serving as the background (Table S13, Zhang et al.^16^). The resulting *P*-values for each enriched (*P* < 0.05) were transformed into *x* = −log_10_(*P*), and these *x*-values for all four groups were subjected to hierarchical clustering (Euclidean distance, Single linkage) using Cluster 3.0 software.^120^ The clustering results were then visualized using Java TreeView software.^121^

## Supplementary Material



## Notes

PARylation stoichiometries for 235 identified proteins calculated from four mix ratios, and functional connections among the PARylated proteins (PDF)
