# Supplementary material for: A Large-Scale Method to Measure the Stoichiometries of Protein Poly-ADP-Ribosylation
Source: ACS Chem Biol. 2026 Feb 26;21(3):527–45. doi: 10.1021/acschembio.5c00817 (PMC13010286; doi:10.1021/acschembio.5c00817)
Supplement: Supplementary file 1 [file cb5c00817_si_001.pdf]

Supporting Information

A large-scale method to measure the stoichiometries of protein Poly-ADP-Ribosylation

Peng Li[1,3], Yajie Zhang[1,3], Chiho Kim[2], Yonghao Yu[1,2*]

[1]Department of Biochemistry, University of Texas Southwestern Medical Center, Dallas, TX 75390, USA.

[2]Department of Molecular Pharmacology and Therapeutics, Columbia University Vagelos College of Physicians and Surgeons, New York, NY 10032, USA

[3]These authors contributed equally to this work

[*]Corresponding author

Email: yy3213@cumc.columbia.edu (Y.Y.)

This file includes:

Supplementary figure: Figure S1

Supplementary tables: Table S1 and S2

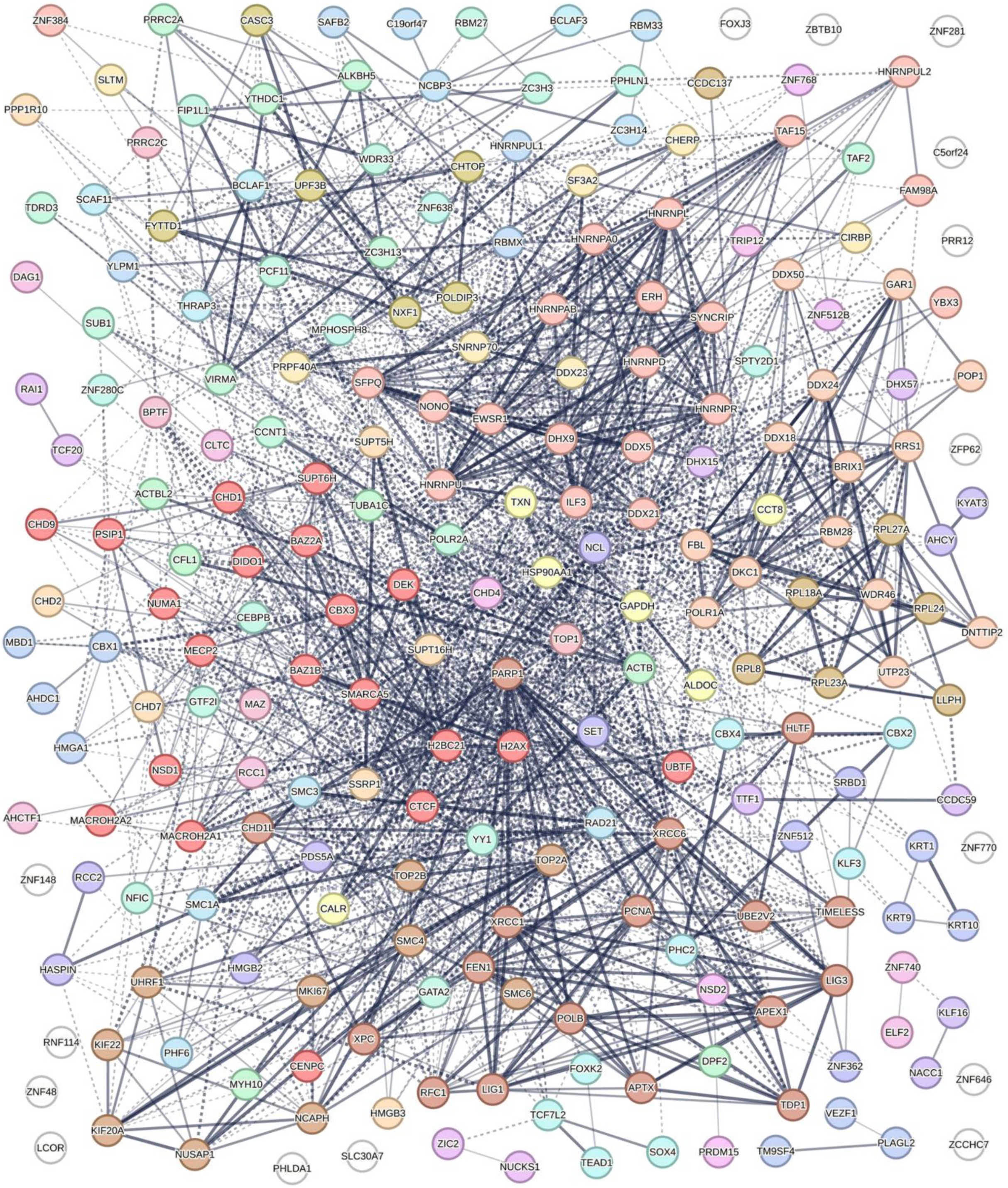

Figure S1. Functional connections among the PARylated proteins.

The network of functional connections among the 235 PARylated proteins identified in PARylation stoichiometry analysis was determined by STRING analysis (STRING Version 11.5).

Table S1. The PARylation stoichiometry for each protein calculated based on results obtained from all four mix ratios.

Table S2. The PARylation stoichiometries of 235 identified proteins.
